# ACACB is a novel metabolism-related biomarker in the prediction of response to cetuximab therapy inmetastatic colorectal cancer: ACACB is a biomarker for CTX therapy in metastatic CRC

**DOI:** 10.3724/abbs.2022121

**Published:** 2022-09-13

**Authors:** Hi-Ju Hong, Yanfei Shao, Sen Zhang, Guang Yang, Hongtao Jia, Xiao Yang, Ling Huang, Shuchun Li, Batuer Aikemu, Luyang Zhang, Junjun Ma, Lu Zang, Jing Sun, Minhua Zheng

**Affiliations:** 1 Department of General Surgery Ruijin Hospital Shanghai Jiao Tong University School of Medicine Shanghai 200025 China; 2 Department of Gastrointestinal Surgery Ruijin Hospital Shanghai Jiao Tong University School of Medicine Shanghai 200025 China; 3 Shanghai Minimally Invasive Surgery Center Ruijin Hospital Shanghai Jiao Tong University School of Medicine Shanghai 200025 China; 4 Wuxi Branch of Ruijin Hospital Wuxi 213021 China

**Keywords:** colorectal cancer, cetuximab, targeted therapy, ACACB, comprehensive analysis

## Abstract

Cetuximab is one of the most valuable targeted therapy monoclonal antibodies in the treatment of metastatic colorectal cancer (CRC). However, the mechanisms affecting cetuximab resistance in CRC treatment remain unclear. Metabolism, especially fatty acid metabolism, has been reported to play an important role in tumor treatment. The correlation between cetuximab resistance and metabolism and whether it can be a new biomarker to evaluate the sensitivity of cetuximab in CRC treatment still need to be further explored. In this study, we perform a comprehensive analysis to confirm the relationship between fatty acid metabolism and cetuximab resistance, and the differentially expressed genes (DEGs) related to cetuximab drug resistance in CRC are screened by bioinformatics technology. We find that acetyl-CoA carboxylase beta (ACACB), ADH1C, CES1, MGLL, FMO5, and GPT are the hub DEGs, and ACACB is the most important biomarker among them. In addition, we systematically analyze the role of ACACB in the tumorigenesis of CRC, including tissue expression, CRC cell growth, cetuximab sensitivity, and potential downstream pathways, by using bioinformatics techniques, *in vitro* experiments and clinical cohort validation. Our results confirm that cetuximab resistance is correlated with metabolism. ACACB can lead to decreased sensitivity to cetuximab in CRC, and its mechanism may be related to EGFR phosphorylation, which could affect the activation of the mTOR/Akt signaling pathway and regulation of CDT1-, cyclin D1-, and p21-related cell cycle modulation.

## Introduction

Colorectal cancer (CRC) is one of the most commonly diagnosed malignancies worldwide. Although the prognosis of CRC patients has been significantly improved with the progress and development of treatment, the prognosis of metastatic CRC (mCRC) patients is still poor, and the five-year survival rate is less than 20% [1]. Several studies have shown that RAS wild-type mCRC patients can benefit from cetuximab therapy in recent years [ 2– 4] . However, owing to cetuximab resistance in mCRC, more than 25% of patients cannot benefit from conversion therapy [5]. Therefore, it is meaningful to explore the potential mechanisms of cetuximab resistance and find more effective biomarkers that can help identify specific patients who are sensitive to cetuximab and individualize their treatment.

CRC is a disease related to certain genes and can even be regarded as a metabolic disease caused by genetic alterations [ 6– 8] . Previous studies have shown that tumor cells are different from normal cells in metabolic networks [ 9, 10] . Tumor cells have many more reactions active in glycolysis, fatty acid synthesis, and glutamine metabolism [ 9, 10] . In addition, the metabolism of the tumor is regarded as a potentially effective target for CRC therapy [11]. The pharmacological mechanism of cetuximab is its unique role as an antibody against epidermal growth factor receptor (EGFR), which also plays an important role in tumor metabolism [12]. Few studies have systematically explored the relationship between cetuximab resistance and metabolism, although some possible mechanisms of cetuximab resistance in CRC have been reported [ 9, 10, 13] .

Acetyl-CoA carboxylase (ACC) is a key and rate-limiting switch in the first step of fatty acid synthesis and metabolism that can catalyze the conversion of acetyl-CoA to malonyl-CoA, playing an important role in the occurrence and development of tumors [ 14, 15] . ACC is divided into two subtypes: acetyl-CoA carboxylase alpha (ACACA) and acetyl-CoA carboxylase beta (ACACB). ACACB is mainly distributed in the outer membrane of mitochondria, and increased ACACB expression can be found in tissues with active oxidation, such as the skeletal muscle, heart and other adipose tissues. The main role of ACACB is to produce malonyl CoA, which is an effective inhibitor of carnitine palmityl transferase (CPT-1) and further effectively inhibits the oxidation of fatty acids [ 16– 18] . However, the role of ACACB in CRC tumorigenesis and cetuximab resistance remains unknown.

In the present study, we explored the correlation between cetuximab resistance and metabolism, and screened the differentially expressed genes (DEGs) related to cetuximab drug resistance in CRC by bioinformatics technology. In addition, we systematically analyzed the role of ACACB in the tumorigenesis of CRC, including tissue expression, CRC cell growth, cetuximab sensitivity, and potential downstream pathways, by using bioinformatics techniques, *in vitro* experiments, and clinical cohort validation.

## Materials and Methods

### Acquisition of data

The Cancer Genome Atlas (TCGA) database covers the RNA-sequencing and clinical data of almost 33 cancer types. TCGA-COAD and TCGA-READ datasets were combined in this study to explore the effect of metabolism-related genes in CRC, and all their corresponding clinical data were downloaded from the University of California Santa Cruz ( https://xenabrowser.net/datapages/). In addition, GSE56386 was used to explore the molecular mechanism of cetuximab drug sensitivity in CRC, and all its corresponding data were downloaded from the Gene Expression Omnibus (GEO) database ( http://www.ncbi.nlm.nih.gov/geo/). Furthermore, the 896 metabolism-related genes were obtained from previously reported papers and searched by the keyword “Metabolism”.

### Screening of Hub genes

The DEGs between the normal ( *n*=51) and tumor samples ( *n*=599) were screened by the R package “limma”, with a cut-off value false discovery rate (FDR)<0.01 and |log 2-fold change (log2FC)|>1. The DEGs between the cetuximab-sensitive ( *n*=4) and cetuximab-nonsensitive ( *n*=4) samples were also screened by the R package “limma”, with a cut-off *P* value<0.05 and |log2FC|>2.5. A Venn diagram of 11 DEGs was visualized by the web tool “Venny” ( https://bioinfogp.cnb.csic.es/tools/venny/). The protein-protein interaction (PPI) networks were constructed by the Search Tool for the Retrieval of Interacting Genes/Proteins (STRING, http://www.string-db.org/). CytoHubba of Cytoscape software (version 3.9.0) was applied to further screen the hub genes of cetuximab drug sensitivity in CRC. In addition, overall survival analysis of the hub genes was performed to explore their prognostic values in CRC patients (TCGA dataset) by the R package “survival”.

### Expression difference and functional analysis of ACACB

The different expression levels of ACACB between the normal and tumor CRC samples were further analyzed in different ways, including the bioinformatics analysis of the TCGA pancancer dataset, Oncomine tool ( https://www.oncomine.org/resource/), CCLE tool ( https://sites.broadinstitute.org/ccle/), and the immunohistochemistry results in CRC. Furthermore, the limma R package was applied to screen the DEGs between the high and low ACACB expression groups in the TCGA dataset. The GO and KEGG analyses were performed and visualized using the OmicShare tools, a free online platform for data analysis ( https://www.omicshare.com/tools). The CTRP- and PRISM-derived drug response data were applied to identify the potential therapeutic agents for CRC with high ACACB expression.

### Cell culture and transfection

CRC cell lines (LoVo, HCT116, RKO, HT29, SW48, SW480, SW620, and NCI-H508) were purchased from the Cell Bank of the Chinese Academy of Sciences (Shanghai, China). All cells were cultured in DMEM (HyClone, Logan, USA) with 10% fetal bovine serum (FBS), 100 U/mL penicillin, and 0.1 μg/mL streptomycin under a humidified atmosphere containing 5% CO _2_ at 37°C. To establish ACACB-knockdown (ACACB-KD) stable cells, cells were seeded in a 6-well plate, cultured for 24 h, transfected with ACACB-KD virus or control virus (Genechem, Shanghai, China), and further treated with puromycin to obtain ACACB-KD stable cell clones. The target and control sequences were as follows: KD-1: 5′-TCCTGACATACACTGAATT-3′; KD-2: 5′-CTCGTAGATGTGGAATTAA-3′; and sh-Control: 5′-TTCTCCGAACGTGTCACGT-3′. Finally, mRNA and protein expression levels were measured to validate the virus transfection efficiency before the subsequent experiments.

### Cell viability assay

After being exposed to gradient concentrations of cetuximab (Merk, Darmstadt, Germany), cell viability was measured using Cell Counting Kit 8 (CCK8; Dojindo Molecular Technologies, Tokyo, Japan) following the manufacturer’s protocol. The IC _50_ value of cetuximab was determined using nonlinear regression.

### Quantitative RT-PCR (qPCR) analysis

Total RNA was isolated from cell lines and tissues using Trizol (Invitrogen, Carlsbad, USA) according to the manufacturers’ instructions. cDNA was synthesized by using a Reverse Transcription kit (Invitrogen). qPCR was performed using SYBR Green PCR Master Mix (Applied Biosystems, Foster City, USA). The sequences of the primers used for qPCR analysis were as follows: *GAPDH* (forward: 5′-TTCAACAGCAACTCCCACTCTT-3′, reverse 5′-TGGTCCAGGGTTTCTTACTCC-3′) and *ACACB* (forward: 5′-TCTCCCGCTGAGTTTGTCAC-3′, reverse 5′-GGACGGGGACGTAATGATCC-3′). In addition, the microsatellite instability (MSI) status of CRC patients was determined by a five-Bethesda marker (NR-24, BAT-25, BAT-26, CAT-25, and MONO-27) panel. The microsatellite instability (MSI) status of CRC patients was determined by a five-Bethesda-marker (NR-24, BAT-25, BAT-26, CAT-25, and MONO-27) panel. Tumors with instability in two or more of the five markers were classified as microsatellite instability-high (MSI-H). Those with one unstable marker were classified as microsatellite instability-low (MSI-L), whereas tumors with all five stable markers were classified as microsatellite stability (MSS).

### Western blot analysis

Western blot analysis was performed as previously described [ 19, 20] . Primary antibodies were purchased as follows: GAPDH (1:5000; Proteintech, Chicago, USA); ACACB (1:1000; Invitrogen); EGFR (1:1000; Cell Signaling Technology, Beverly, USA); p-EGFR (Tyr 1068) (1:1000, Cell Signaling Technology); mTOR (1:1000; Cell Signaling Technology); p-mTOR (Ser 2448) (1:1000; Cell Signaling Technology); p21 (1:1000; Abcam, Cambridge, UK); CDT1 (1:1000; Abcam); Akt (1:1000; Cell Signaling Technology); and p-Akt (Ser 473) (1:1000; Cell Signaling Technology). The following HRP-conjugated anti-rabbit or anti-mouse IgG secondary antibodies (1:5000; Proteintech) were used.

### Immunohistochemistry (IHC)

All formalin-fixed, paraffin-embedded tumor tissue sections were acquired from Ruijin Hospital (Shanghai, China). IHC was performed according to standard procedures. Each slide was scored by two independent pathologists using a semiquantitative method according to the German semi-quantitative scoring system [ 20, 21] . Tumors with instability at two or more of the five markers were classified as microsatellite instability-high (MSI-H). Those with one unstable marker were classified as microsatellite instability-low (MSI-L), whereas tumors with all five stable markers were classified as microsatellite stability (MSS). IHC in this study included EGFR, FGF2, and ACACB.

### Patients and tissue samples

To investigate the correlation between ACACB level and sensitivity to clinical application of cetuximab and clinical prognosis of patients, we collected and analyzed the data of mCRC patients treated with cetuximab at Shanghai Minimally Invasive Surgery Center, Ruijin Hospital Affiliated to Shanghai Jiao Tong University School of Medicine from November 2012 to September 2019. This study was designed as a single-center retrospective cohort analysis, and patients with CRC who underwent surgery and received cetuximab were included in the study. Clinical data, pathological results, imaging data, follow-up efficacy analysis, laboratory-related experimental results, and pathology results were collected and analyzed. This study was approved by the Ethics Committee of Ruijin Hospital, School of Medicine, Shanghai Jiao Tong University, and all of the enrolled patients signed informed consent forms.

### Efficacy evaluation of cetuximab

In this study, the efficacy evaluation (RECIST) criteria version 1.1 guideline for solid tumors was used, and whole-body CT images were taken before treatment as the baseline, and the images captured during cetuximab treatment were compared with the baseline [22]. The sensitivity of cetuximab treatment was evaluated according to the evaluation results. The response group included complete response (CR), partial response (PR), and stable disease (SD) cases after evaluation; the nonresponse group included progressive disease (PD) patients after evaluation.

### Clinical follow-up

The enrolled patients were followed up through outpatient service, telephone, WeChat app, etc. The enrolled patients were required to complete the follow-up project according to the prescribed time. Patients enrolled in the study were required to complete the baseline assessment of tumor indicators by whole-body chest, abdomen, and pelvis enhanced CT or MR before treatment. During treatment, patients were reexamined for tumor indicators every 3 months by whole-body chest, abdomen, and pelvis enhanced CT or MR, and every 6 months in patients who showed complete response without tumor recurrence trends during the follow-up.

### Statistical analysis

In this study, R software (version: 3.6.3) was used to analyze the number of surviving cells in the cell line and draw the graph. All the results were calculated by R software. Data are expressed as the mean±SD. Two-sided unpaired Student’s *t* test, two-way ANOVA, the Kaplan-Meier method, the χ ^2^-test, and the log-rank test were used to analyze the data. *P*<0.05 was considered statistically significant.

## Results

### *ACACB* is a co-hub gene related to metabolism as well as cetuximab resistance in CRC

**Figure 1:**
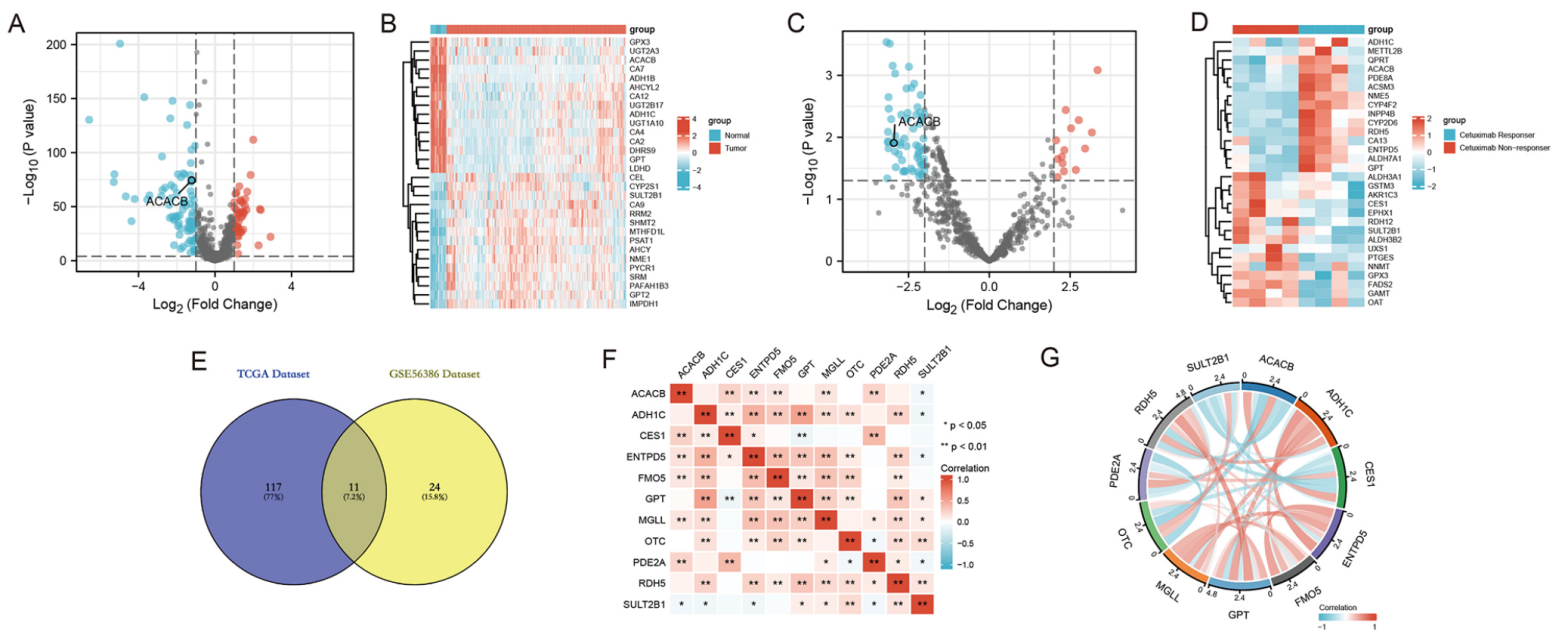
Figure 1 Identification and analysis of the differentially expressed genes related to metabolism and cetuximab resistance in CRC (A,B) Volcano map and Heatmap of metabolism-related differentially expressed genes (DEGs) between the tumor and normal colon tissues in the TCGA datasets. (C,D) Volcano map and heatmap of the metabolism-related DEGs between the cetuximab responders and nonresponders in the GSE56386 dataset. (E) Venn diagram identifying the 11 co-DEGs between the TCGA and GSE56386 datasets. (F,G) Correlation network plots of the 11 DEGs.

**Figure 2:**
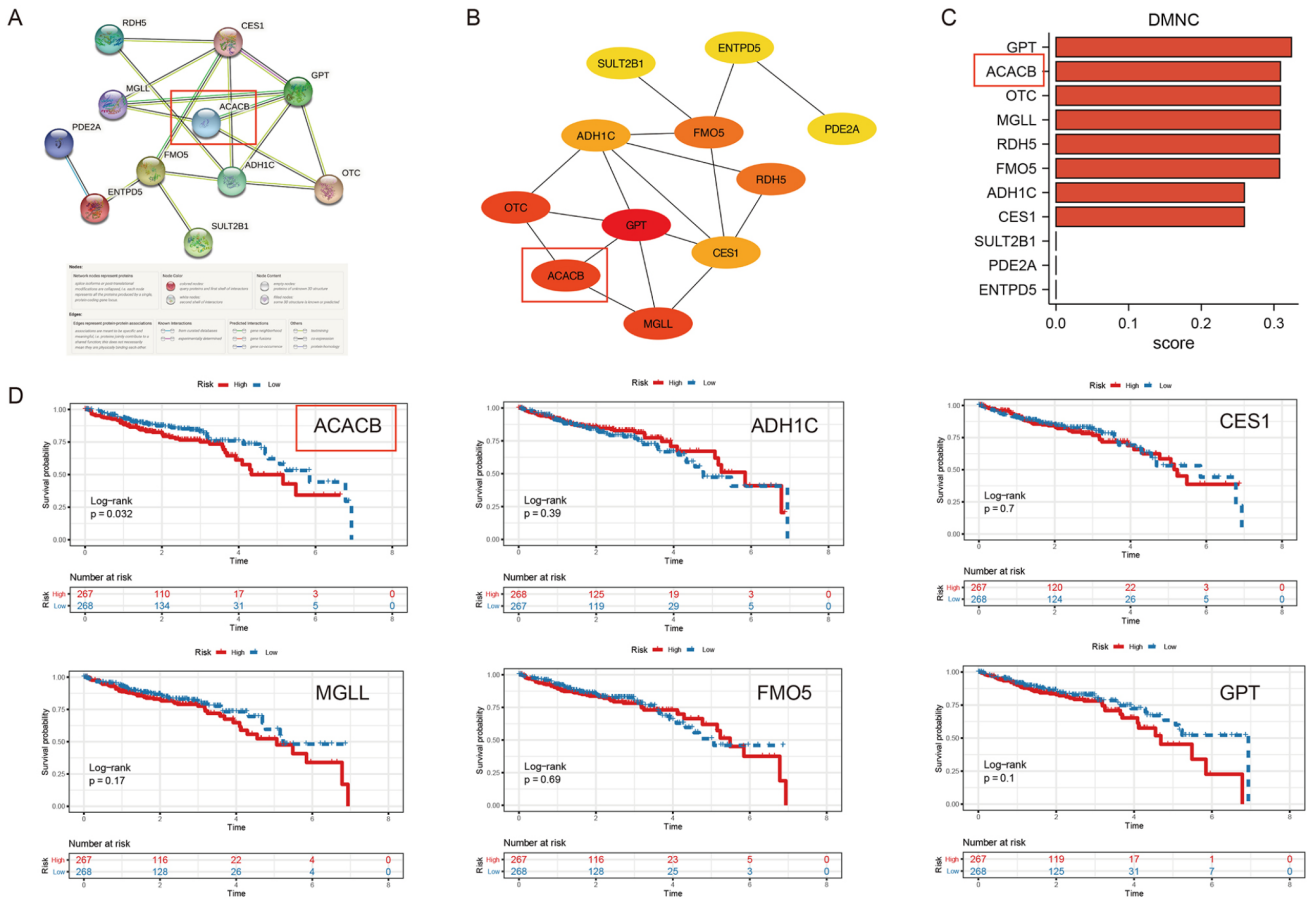
Figure 2 Screening of the co-hub genes related to metabolism and cetuximab resistance in CRC (A,B) The PPI network plots of the 11 co-DEGs by STRING (A) and Cytoscape (B). (C) The bar plot showing the rank of 11 co-DEGs by the DMNC algorithm of Cytoscape. (D) Kaplan-Meier survival plots of the 6 co-hub DEGs in TCGA datasets.

A total of 128 metabolism-related genes were screened between the normal and tumorous tissues of CRC patients in the TCGA dataset and visualized by the volcano plot and heatmap ( Figure 1A,B). Thirty-five metabolism-related genes were also screened between the cetuximab-sensitive and nonsensitive samples in GSE56386 and visualized by the volcano plot and heatmap ( Figure 1C,D). Eleven of them were both metabolism-related DEGs and cetuximab drug resistance-associated DEGs, visualized in the Venn diagram ( Figure 1E). The correlation among these 11 gene expressions is visualized ( Figure 1F,G). The PPI networks indicated the following hub genes: *ACACB*, *ADH1C*, *CES1*, *MGLL*, *FMO5*, and *GPT* ( Figure 2A–C). In addition, the overall survival analysis of these 6 hub genes showed that only *ACACB* is related to the prognosis of CRC ( *P*=0.032), as shown in Figure 2D.

### ACACB is highly expressed in CRC

**Figure 3:**
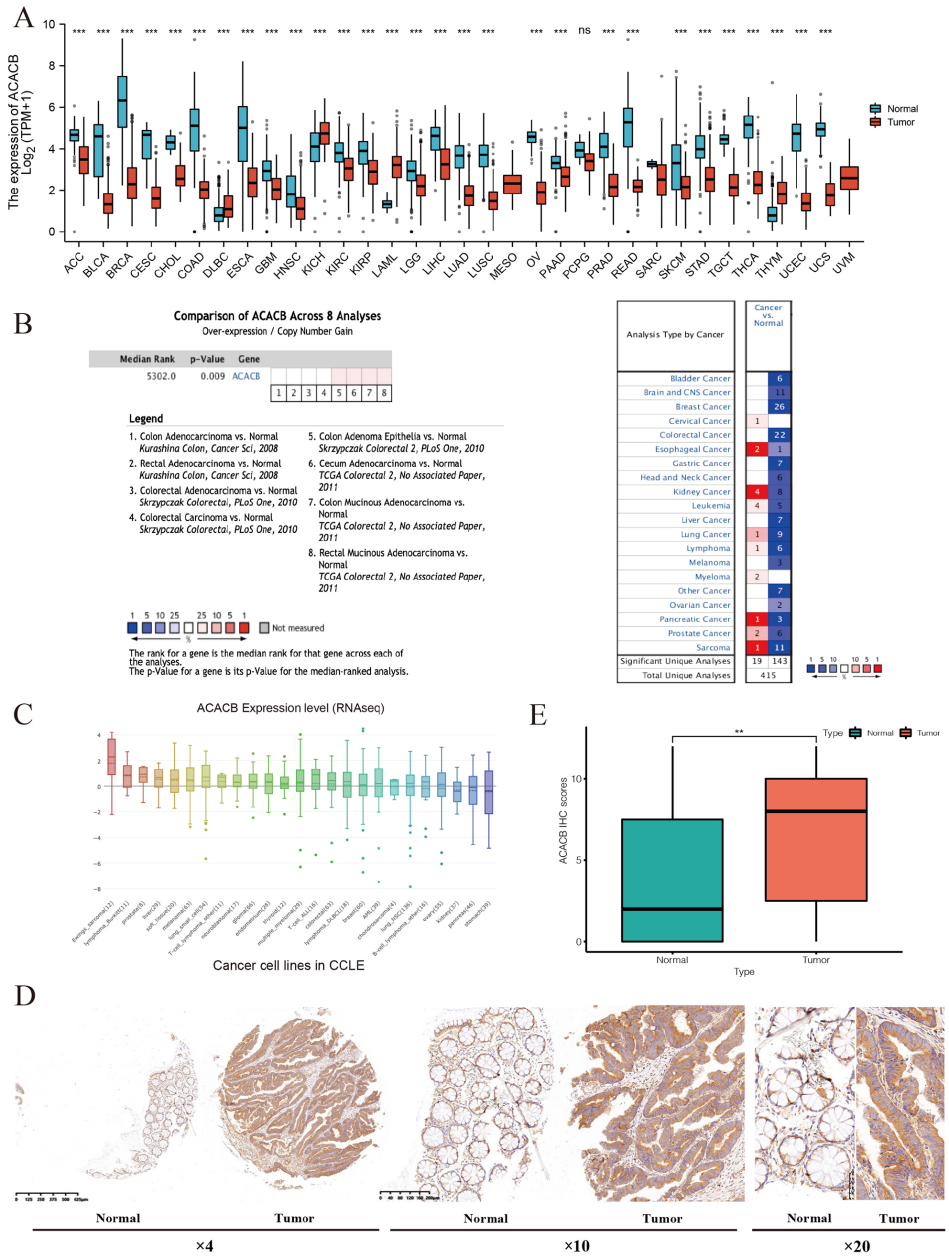
Figure 3 Expression of ACACB in CRC (A) The ACACB expression levels between normal and tumor tissues in the TCGA pancancer datasets. (B) The ACACB expression levels between the normal and tumor tissues in CRC determined by the Oncomine tool. (C) ACACB expression levels in different kinds of cancer cell lines. (D) Representative immunohistochemistry images of ACACB in CRC and corresponding normal tissues from our center (magnification: ×4, ×10, and ×20, respectively). (E) The semi-quantitative immunohistochemistry results of ACACB in paired CRC tissues from our center. Data are presented as the mean±SD. Unpaired Student’s t test, ** P<0.01.

The pancancer analysis in TCGA datasets indicated lower expression of ACACB in tumor tissue, including colon and rectal cancers ( Figure 3A). However, the data from different cancer centers by the Oncomine tool showed opposite results between CRC tissue and normal colon tissue, showing decreased ACACB expression in tumor tissues ( Figure 3B,C). In addition, based on the CCLE database, ACACB is expressed in almost all cancer cell lines, including CRC cell lines ( Figure 3D). Considering the controversial results, to further confirm the expression of ACACB in CRC, we collected 74 paired CRC tumors and normal tissues from our center for immunohistochemistry analysis. The results indicated that ACACB was significantly overexpressed in CRC tissues ( Figure 3E,F).

### ACACB promotes CRC cell growth and cetuximab resistance in CRC cell lines

**Figure 4:**
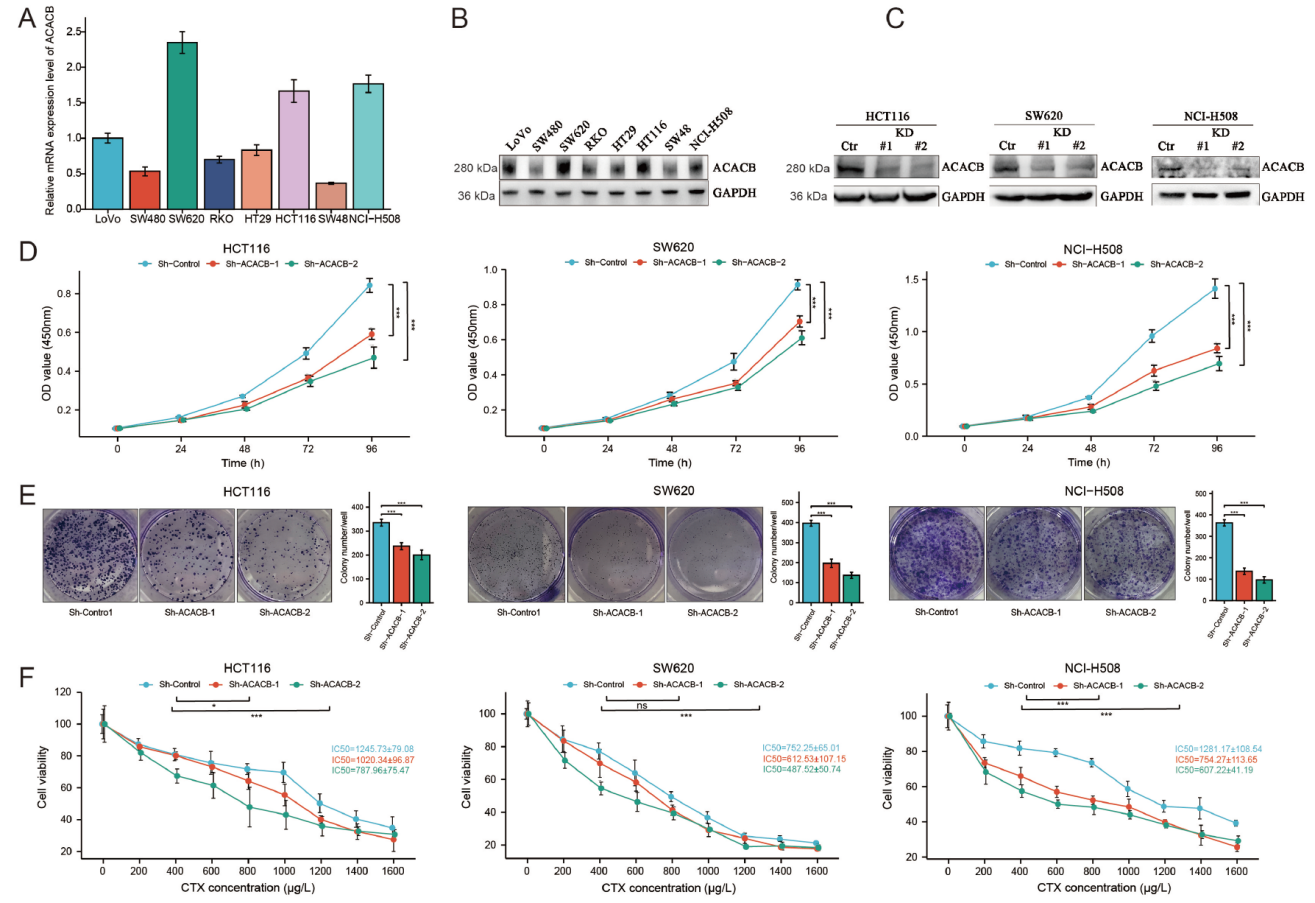
Figure 4 ACACB promotes CRC cell growth and cetuximab resistance in CRC cell lines (A) The relative mRNA expression levels of ACACB in different CRC cell lines were measured by real-time PCR and presented as fold changes relative to LoVo cell lines. (B) Western blot analysis of ACACB in different CRC cell lines. GAPDH served as a loading control. (C) Verification of the knockdown efficiency of ACACB in HCT116, SW620, and NCI-H508 cell lines. (D) The effect of ACACB on cell proliferation was measured in vitro by CCK8 assay in HCT116, SW620, and NCI-H508 cell lines. (E) Representative images showing the results of the colony formation assay in control and ACACB-knockdown HCT116, SW620, and NCI-H508 cell lines. (F) Line charts showing the viability and IC 50 values of cetuximab in the HCT116, SW620, and NCI-H508 cell lines. Experiments were repeated three times in each group. Data are presented as the mean±SD. * P<0.05, *** P<0.001, and ns, not significant.

Further analysis of the mRNA and protein expression levels of ACACB in LoVo, SW480, SW620, RKO, HT29, HCT116, SW48, and NCI-H508 cell lines was performed. The results indicated that the HCT116, SW620, and NCI-H508 cell lines had the highest expression levels of ACACB ( Figure 4A,B). In addition, NCI-H508 is a KRAS-WT and BRAF-WT CRC cell line. Based on these results, to further explore the effect of ACACB on CRC cell growth and cetuximab resistance in CRC cell lines, two shRNAs were synthesized to knock down *ACACB*, and stable knockdown cell lines (KD#1 and KD#2) were generated according to the manual in HCT116, SW620 and NCI-H508 cell lines ( Figure 4C). CCK8 and colony formation results showed that the knockdown of *ACACB* decreased cancer cell proliferation and blocked colony formation in the HCT116, SW620, and NCI-H508 cell lines ( Figure 4D,E). To confirm the correlation between ACACB expression and cetuximab sensitivity, the IC _50_ values for cetuximab were measured, and the results also revealed that the IC _50_ values of cetuximab in the HCT116, SW620, and NCI-H508 cell lines were all decreased after the knockdown of *ACACB* ( Figure 4F), which indicated increased sensitivity to cetuximab. These data revealed that the variation in ACACB level had an obvious effect on CRC cell growth and sensitivity to cetuximab.

### Functional enrichment and pathway analysis of ACACB in CRC

**Figure 5:**
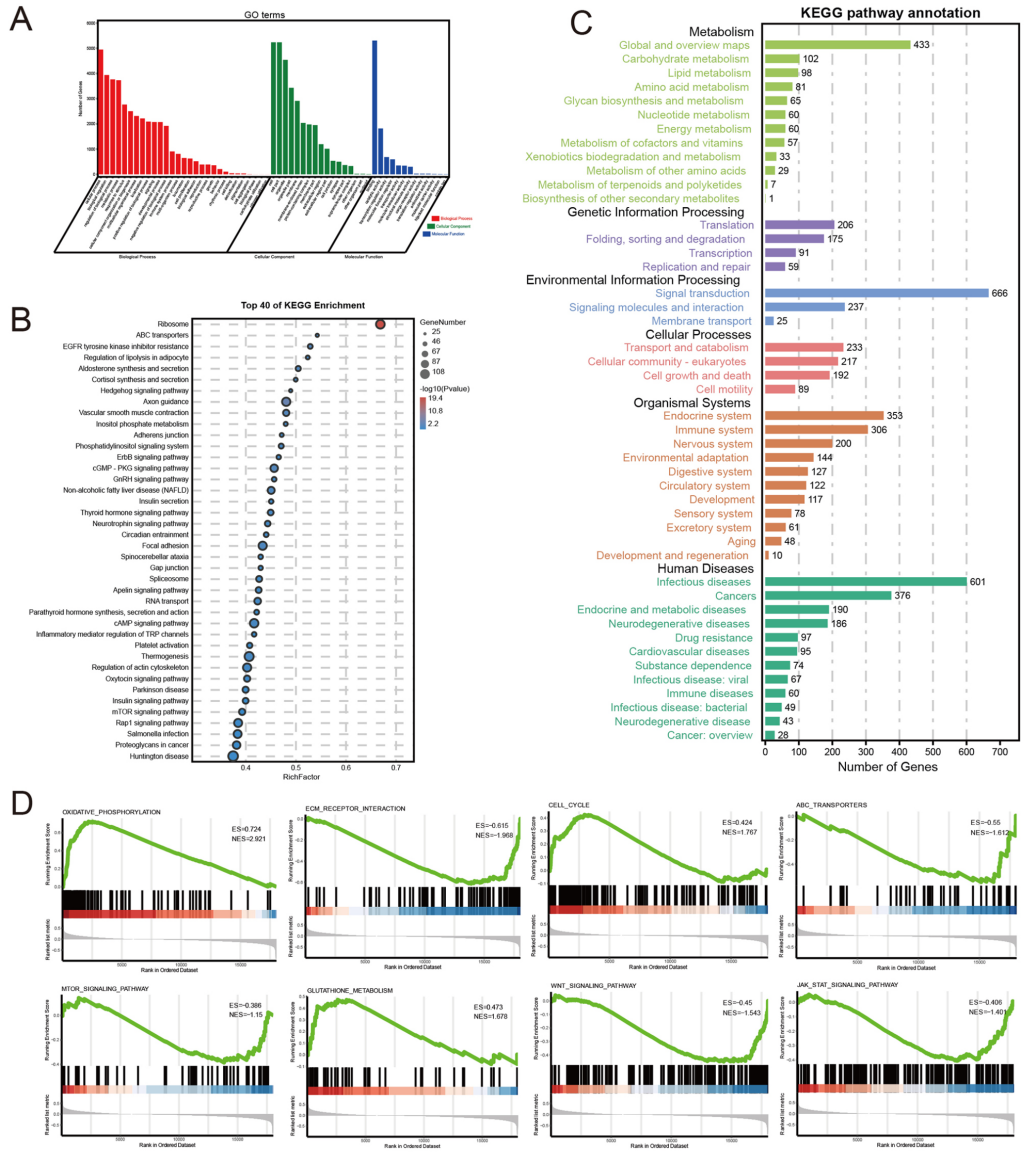
Figure 5 Functional enrichment and pathway analysis of ACACB in CRC (A) GO enrichment analysis of the DEGs between the high and low ACACB expression groups in the TCGA datasets. (B) The top 40 enriched KEGG pathways of the DEGs between the high and low ACACB expression groups in the TCGA datasets. (C) All the KEGG pathway annotations. (D) GSEA enrichment analysis between the high and low ACACB expression groups.

﻿To better understand the underlying mechanism of ACACB in tumorigenesis and cetuximab sensitivity in CRC, DEG analysis of the TCGA dataset was performed between the high ( *n*=300) and low ( *n*=299) ACACB expression subcohorts. Based on these DEGs, GO and KEGG pathway enrichment analyses were further performed. Cell Component (CC), Biological Process (BP), and Molecular Function (MF) were all covered in the GO function analysis. The results showed that the organelle and membrane were enriched for CC; biological regulation, metabolic system, and immune system for BP; catalytic and transcription regulator activity for MF ( Figure 5A). In addition, the results of the KEGG pathway analysis indicated that ABC transporters, EGFR tyrosine kinase inhibitor resistance, focal adhesion, mTOR signaling pathway, etc., were enriched ( Figure 5B,C). Furthermore, oxidative phosphorylation, ECM receptor interaction, cell cycle, ABC transporters, mTOR signaling pathway, glutathione metabolism, Wnt signaling pathway, and JAK stat signaling pathway were also enriched by GSEA ( Figure 5D). These results further confirmed that ACACB is correlated with both tumor metabolism and cetuximab resistance.

### Downstream pathway analysis of ACACB in CRC cell lines *in vitro*

**Figure 6:**
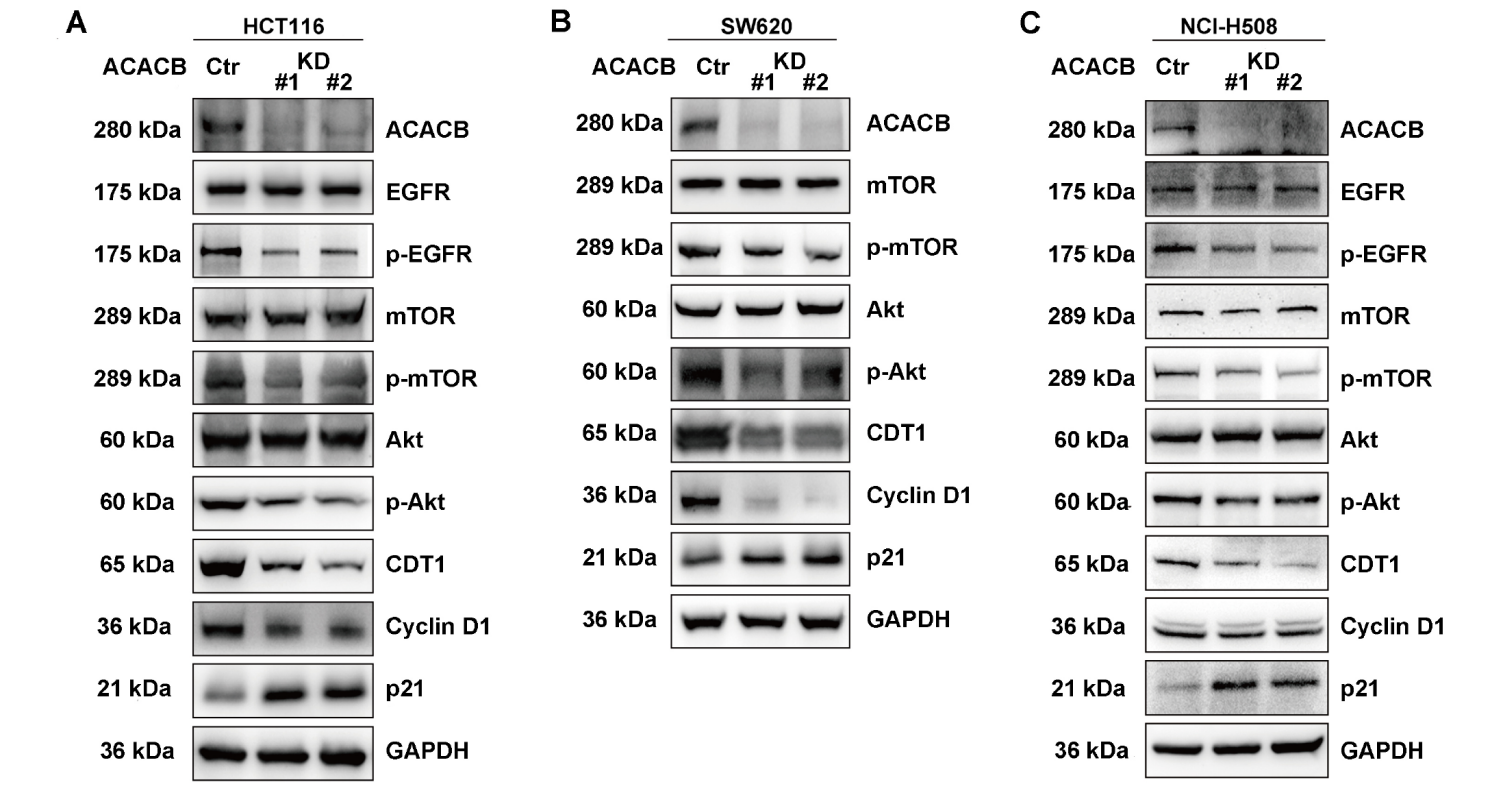
Figure 6 Downstream pathway analysis of ACACB in CRC cell lines (A) Western blot analysis of ACACB, EGFR, p-EGFR, mTOR, p-mTOR, Akt, p-Akt, CDT1, cyclin D1, and p21 in the HCT116 cell line. (B) Western blot analysis of ACACB, mTOR, p-mTOR, Akt, p-Akt, CDT1, cyclin D1, and p21 in the SW620 cell line. (C) Western blot analysis of ACACB, EGFR, p-EGFR, mTOR, p-mTOR, Akt, p-Akt, CDT1, cyclin D1, and p21 in the NCI-H508 cell line. GAPDH served as a loading control.

Based on the results of the functional enrichment and pathway analysis of ACACB, further explorations of related signaling pathways were performed to confirm the potential mechanisms between ACACB expression and cetuximab sensitivity in different CRC cell lines. Western blot analysis showed that p-EGFR, p-mTOR, p-Akt, CDT1, and cyclin D1 were downregulated and p21 was upregulated in HCT116 cells after *ACACB* knockdown ( Figure 6A). In SW620 cells, p-mTOR, p-Akt, CDT1, and cyclin D1 were downregulated, while p21 was upregulated. EGFR was not expressed in SW620 cells ( Figure 6B). In NCI-H508 cells, p-EGFR, p-mTOR, p-Akt, and CDT1 were downregulated, while p21 was upregulated ( Figure 6C). These results suggested that the knockdown of *ACACB* could inhibit EGFR-related pathways.

### ACACB expression and prognostic analysis of mCRC patients treated with cetuximab

**Figure 7:**
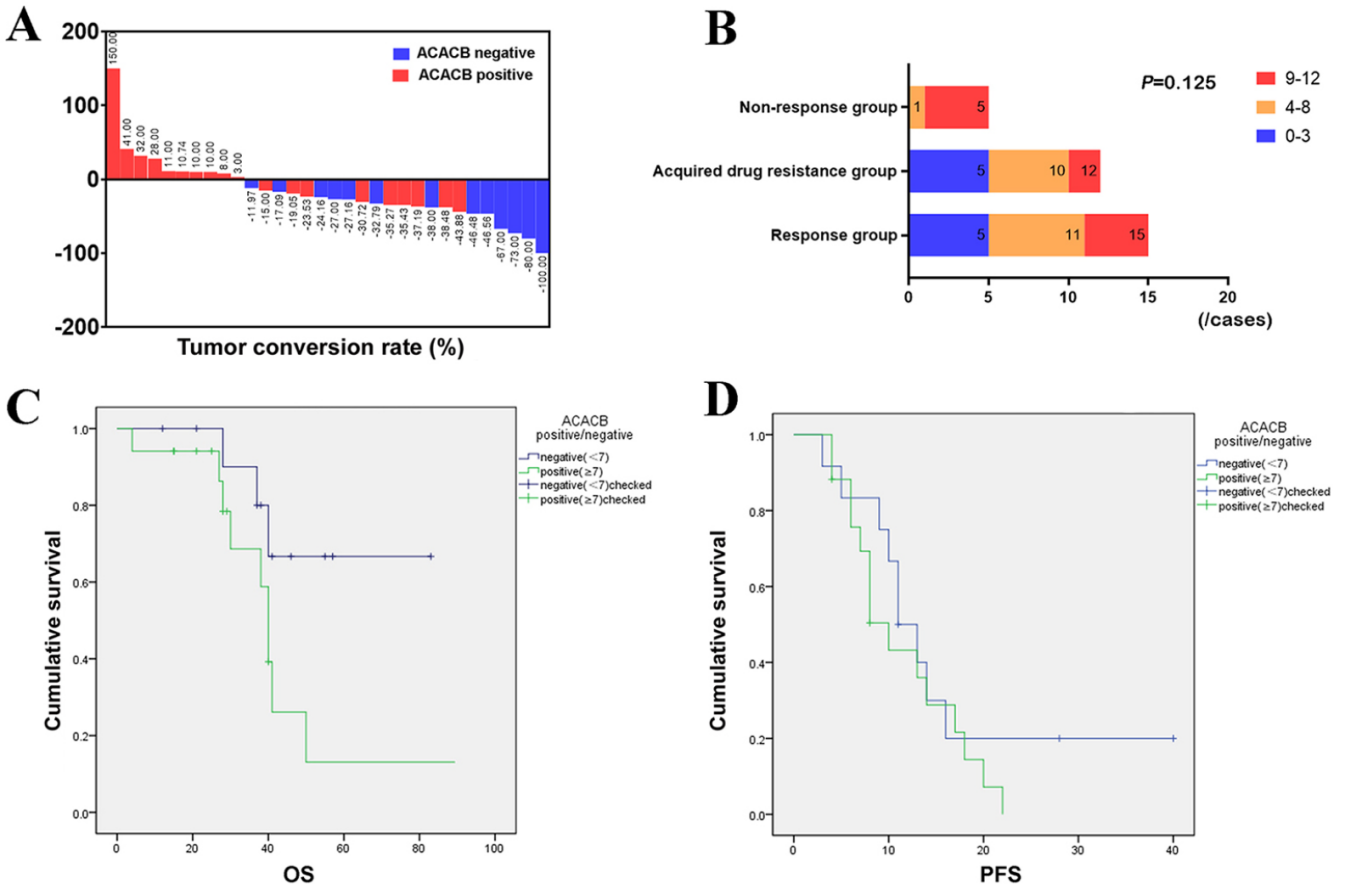
Figure 7 ACACB expression and prognostic analysis of mCRC patients treated with cetuximab (A) Conversion rates in ACACB-negative and ACACB-positive groups. (B) The proportions of ACACB expression in the cetuximab nonresponse, acquired drug resistance, and response groups. (C) Overall survival curves in ACACB-negative and ACACB-positive groups. (D) Progression-free survival curves in ACACB-negative and ACACB-positive groups.

The conversion rate of the tumor was analyzed, and the results showed that the ACACB-negative group showed better tumor regression after cetuximab treatment ( Figure 7A). According to the IHC score of ACACB expression, we divided them into three groups: 0–3 points, 4–8 points, and 9–12 points. In addition, histograms were drawn and compared for the number and proportion of cases in the response group-sustained effective group, response group-acquired drug resistance group, and nonresponse group. Figure 7B shows that with the increase in the ACACB immunohistochemical score, that is, the increase in ACACB expression, the proportion of the corresponding nonresponsive group is increased. These results suggested that tumors with higher ACACB expression have a higher possibility of cetuximab resistance.

In the cohort, there were 12 patients in the ACACB-negative group; among them, 6 patients died during the follow-up period. There were a total of 17 patients in the ACACB-positive group, and among them, 7 patients died. The mean survival time of the ACACB-negative group was 41.25±18.32 months, and the median survival time was 39 months (12 to 83 months). The mean survival time of the ACACB-positive group was 31.24±13.71 months, and the median survival time was 29 months (4 to 60 months; *P*=0.037). The mean progression-free survival (PFS) of the ACACB-negative group was 14.25±10.21 months, and the median PFS was 11 months (3–40 months). The mean PFS of the ACACB-expressing group was 10.41±5.83 months, and the median PFS was 8 months (4 to 22 months; *P*=0.321) ( Figure 7C,D).

The above results suggested that compared with the ACACB-positive group, the survival benefit of the ACACB-negative group was statistically significant. Moreover, a trend of benefit for PFS was found in the ACACB-negative group. This result showed that ACACB could be an indicator to evaluate cetuximab resistance and assist physicians in assessing clinical outcomes.

### ACACB is positively correlated with cetuximab resistance

A total of 32 valid samples were well preserved after the screening, which were from 29 patients from the sample database in our center. Among them, the specimens of 26 patients were from primary lesions, and the specimens of 3 patients were from both primary and metastatic lesions. The 32 samples were divided into the ACACB-positive group and ACACB-negative group according to IHC, and there were 13 cases in the ACACB-negative group and 19 cases in the ACACB-positve group. The clinical demographics of these two groups are shown in Table 1.

**Table:** **Table 1** Clinical characteristics of patients

	ACACB negative ( *n*=12)	ACACB positive ( *n*=17)	*P*
Gender			0.945
Male	10	14	
Female	2	3	
Age (years)	59.00±10.65	60.59±12.38	0.722
BMI (kg/m ^2^)	21.86±3.48	24.04±3.87	0.132
Primary tumor location			0.353
Left colon	9	15	
Right colon	3	2	
Primary tumor location			0.876
Colon	6	9	
Rectum	6	8	
Tumor differentiation			0.550
High	0	0	
Moderate	7	8	
Poor	5	9	
Pathological T stage			0.640
T1	0	0	
T2	0	1	
T3	3	3	
T4	9	13	
Pathological N stage			0.196
N0	2	7	
N1	3	1	
N2	7	9	
Pathological M stage			0.269
M0*	2	6	
M1 ^#^	10	11	
Tumor sage			0.507
I	0	0	
II	1	2	
III	1	4	
IV	10	11	

BMI: body mass index.

*Without metastasis or heterochronous metastasis in the initial evaluation.

^#^With metastasis in the initial evaluation.

The IHC results showed that in the ACACB-negative group, FGF2 was not expressed in 10 cases and was expressed in 3 cases; in the ACACB-positive group, FGF2 was not expressed in 5 cases and was expressed in 14 cases ( *P*=0.005). The status of EGFR, MSI, KRAS, NRAS, PIK3CA2, and BRAF was not significantly different between the two groups ( Table 2).

**Table:** **Table 2** Immunohistochemical analysis data of patients

		ACACB negative ( *n*=13)	ACACB positive ( *n*=19)	*P*
EGFR	Expressed	10	13	0.599
	Non-expressed	3	6	
MSI	MSI-H*	1	1	0.751
	MSI-L ^#^	2	5	
	MSS ^@^	10	13	
KRAS	Mutation	2	2	0.683
	Wild-type	11	17	
NRAS	Mutation	1	2	0.787
	Wild-type	12	17	
PIK3CA20	Mutation	0	0	–
	Wild-type	13	19	
BRAF	Mutation	0	0	–
	Wild-type	13	19	
FGF2	Expressed	3	14	0.005
	Non-expressed	10	5	

*Micro satellite instability-high.

^#^Micro satellite instability-low.

^@^Micro satellite stability.

–: Data not available.

These results suggested that there were significant differences in some part of IHC results between the ACACB-negative group and ACACB-positive group. FGF2, which is associated with immune infiltration, may have a certain correlation with ACACB.

### Identification of potential therapeutic agents for CRC with high ACACB expression

**Figure 8:**
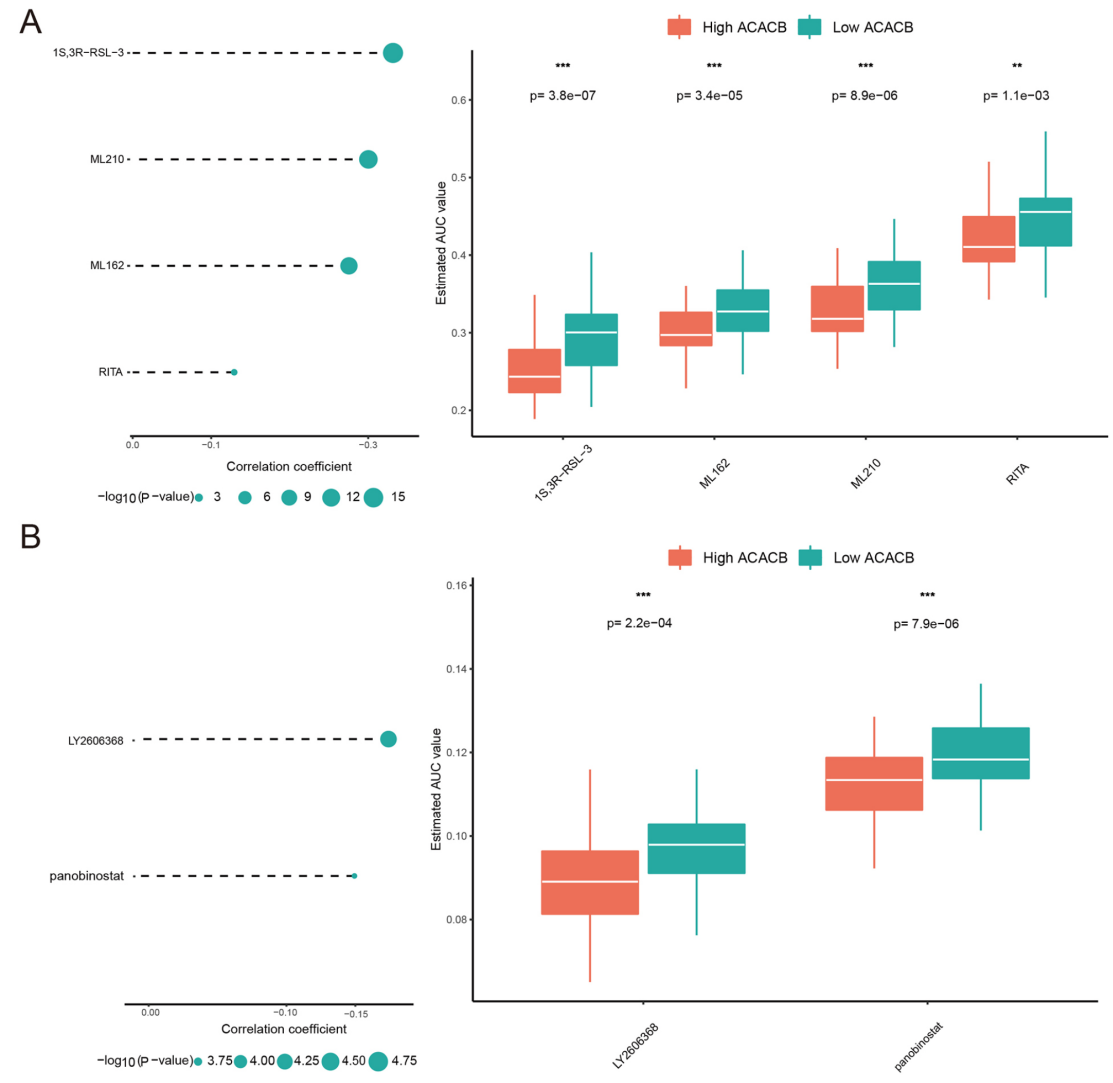
Figure 8 Identification of potential therapeutic agents for CRC with high ACACB expression (A) The differential drug response and correlation analysis of four CTRP-derived compounds. (B) The differential drug response and correlation analysis of two PRISM-derived compounds.

Furthermore, two different methods based on the CTRP and PRISM datasets were applied to screen potential therapeutic agents with higher drug sensitivity in CRC patients with high ACACB expression. Drug response analysis between samples with high ACACB expression (top 10%) and samples with low ACACB expression (bottom 10%) was performed to identify key compounds with lower AUC values (log2FC>0.10). The correlation analysis between the AUC value and ACACB expression level was used to further screen compounds with negative correlation coefficients based on the CTRP ( Figure 8A) and PRISM datasets ( Figure 8B). Finally, four CTRP-derived compounds (including 1S,3R-RSL-3, ML210, ML162, and RITA) and two PRISM-derived compounds (including LY2606368 and panobinostat) were identified as potential therapeutic agents for CRC.

## Discussion

Cetuximab is the most valuable EGFR-related targeted drug for the treatment of CRC. Cetuximab could bind to EGFR and play a role in blocking the binding sites between EGFR and endogenous ligands, thereby reducing abnormal intracellular signaling activation and transduction, inhibiting cell growth, inducing tumor cell apoptosis, and reducing the number of matrix metalloproteinases and VEGF [23]. CELIM, OPUS, and APEC studies have been the most classic phase II clinical studies that can evaluate the efficacy of cetuximab [ 2, 3, 24] . In China, the phase IV RCT BELIEF study showed that the R0 resection rate, objective response rate, overall survival rate, and progression-free survival (PFS) rate were significantly increased in patients with newly diagnosed unresectable KRAS wild-type liver-limited disease (LLD) treated with FOLFOX or FOLFIRI combined with cetuximab [ 25, 5] . However, some patients still have drug resistance before cetuximab application or acquire drug resistance after cetuximab application [ 24, 4] . In the COIN study, the results suggested that cetuximab combined with oxaliplatin did not increase the resection rate of LLD patients [3]. Therefore, identifying specific patients sensitive to cetuximab and individualizing the treatment can not only maximize the clinical efficacy of antitumor therapy but also enable patients to gain more benefits from treatment. More precise indicators are needed to evaluate cetuximab sensitivity, as cetuximab resistance can be found in some CRC patients with RAS wild-type.

It was also found that ACC allosteric inhibitors could restore drug sensitivity to squamous cell carcinoma of the head and neck that had developed cetuximab resistance, which further confirmed that ACC phosphorylation plays a key role in cetuximab resistance [26]. In addition, when being treated based on the Warburg effect, cancer cells may shift their metabolic pathway from glycol-dependent to adipogenesis by phosphorylation of acetyl-CoA carboxylase (ACC) [27]. ACC has been divided into two subtypes, ACACA and ACACB. ACACB has been studied in metabolic syndrome, obesity, and diabetes-related diseases [28]. Meanwhile, ACACB is also a target of metformin, which has also been reported to have a certain inhibitory effect on CRC [29]. In Li’s study, ACACB was found to be highly expressed in laryngocarcinoma and even related to tumor stage and degree of laryngocarcinoma cell differentiation [30]. Moreover, Valvo *et al*. [31] demonstrated that BRAFV600E can downregulate ACACB level and sh-ACACB could increase tumor growth and vemurafenib resistance. ACACB may be a new treatment strategy for overcoming resistance to BRFRV600E inhibitors in papillary thyroid carcinoma. Although some cellular studies have confirmed the correlation between ACC and cetuximab resistance, no further study has been conducted to explore the correlation between ACACB expression and the development of CRC. In addition, whether ACACB plays a key role in mechanisms related to cetuximab resistance remains to be further explored. Therefore, this study was the first to systematically analyze the role of ACACB in the tumorigenesis and cetuximab resistance of CRC.

As a member of the fibroblast growth factor family, which interacts with fibroblast growth factors to activate distinct intracellular signaling PI3K/Akt pathways, FGF2 can promote mitosis and vascular activity and is often associated with tumor growth to a certain extent in the field of oncology and has also been regarded as a tumor risk factor [ 32, 33] . In breast cancer, FGF2 is also positively correlated with epidermal growth factor (EGF) and insulin-like growth factor (IGF) [34]. In addition, Nguyen’s study [35] showed that high expression of FGF2 in the serum of patients with tumor metastasis is correlated with its clinical status, the extent of disease, and mortality risk. Our previous study demonstrated a certain correlation between FGF2 and ACACB [36], but the specific correlation between them needs to be further studied. Moreover, Marshall *et al*. [37] showed that FGFR2 and FGFR3 are commonly expressed in head and neck squamous cell carcinoma (HNSCC) cells and are activated by autocrine FGF2. In addition, the FGFR/Akt/SOX2 signaling axis has been reported to regulate pancreatic cancer stemness properties, which means that FGFR could be a therapeutic target for aggressive cancers [ 38– 40] . Aytatli *et al*. [40] suggested that AZD4547, an orally bioavailable FGFR inhibitor, could inhibit FGFR and its downstream targets in recombinant FGF2 to treat HNSCC cells.

Based on bioinformatics and our comprehensive analysis, we showed that the mechanism of cetuximab resistance is correlated with metabolism and that ACACB plays a key role. ACACB expression was high in tumor tissues, and different expression levels of ACACB could be found in different tumor cell lines. Moreover, ACACB is an independent prognostic factor. In addition, the *in vitro* results showed that knockdown of *ACACB* could decrease CRC cell proliferation and cetuximab resistance in different CRC cell lines. Thus, ACACB expression could promote CRC cell growth and cetuximab resistance *in vitro*. Meanwhile, based on the functional enrichment and pathway analysis of ACACB, we found that many classic pathways in CRC tumorigenesis (such as EGFR tyrosine kinase inhibitor resistance, focal adhesion, and the mTOR signaling pathway) may be downstream pathways of ACACB in CRC. After further investigation, we found that EGFR phosphorylation could affect the activation of the mTOR/Akt signaling pathway, and regulation of CDT1-, cyclin D1-, and p21-related cell cycle modulation could be the potential mechanism of ACACB in CRC. To make the study more scientific and persuasive, we further utilized our own CRC clinical cohorts for validation and found that lower expression of ACACB showed higher sensitivity to cetuximab. Moreover, ACACB expression may change from low to high in cetuximab-sensitive patients who acquire resistance after treatment with cetuximab ( Supplementary Figure S1). In addition, IHC statistical results suggested that ACACB might be correlated with FGF2. The results of our study suggest that the expression of ACACB is correlated with FGF2, which may be related to the mechanism of metabolism leading to cetuximab resistance. These results suggest that FGF2 may be a key factor in addressing cetuximab resistance in collaboration with ACACB. In conclusion, we believe that ACACB could be a novel metabolism-related biomarker in the prediction of the response to cetuximab therapy in CRC. Finally, we identified six compounds (1S,3R-RSL-3, ML210, ML162, RITA, LY2606368, and panobinostat) as potential therapeutic agents for CRC patients with high ACACB expression, which needs to be further verified.

According to this study, we believe that ACACB can be regarded as an effective biomarker for evaluating cetuximab resistance, which can help clinicians further evaluate the use of cetuximab-targeted therapy for CRC patients. With a more accurate assessment of resistance, patients can be more accurately treated with translational therapy, leading to better clinical benefits for patients.

The mechanism of cetuximab resistance is correlated with metabolism, and ACACB plays a key role. ACACB could regulate the sensitivity of CRC cells to cetuximab, which might be related to EGFR phosphorylation, the activation of the mTOR/Akt signaling pathway, and the regulation of CDT1, cyclin D1, and p21 which are related to cell cycle modulation. Patients with higher expression of ACACB showed a worse clinical prognosis, which is consistent with the results of bioinformatics analysis. Cetuximab-sensitive patients who get acquired resistance after cetuximab administration may have increased expression of ACACB. ACACB could be a novel biomarker in the prediction of response to cetuximab therapy in CRC.

## Supplementary Data

Supplementary data is available at *Acta Biochimica et Biophysica Sinica* online.
